# Proteome from patients with metabolic syndrome is regulated by quantity and quality of dietary lipids

**DOI:** 10.1186/s12864-015-1725-8

**Published:** 2015-07-08

**Authors:** Oriol Alberto Rangel-Zúñiga, Antonio Camargo, Carmen Marin, Patricia Peña-Orihuela, Pablo Pérez-Martínez, Javier Delgado-Lista, Lorena González-Guardia, Elena M. Yubero-Serrano, Francisco J. Tinahones, María M. Malagón, Francisco Pérez-Jiménez, Helen M. Roche, José López-Miranda

**Affiliations:** Lipids and Atherosclerosis Research Unit, IMIBIC/Reina Sofia University Hospital, University of Cordoba, Av. Menendez Pidal s/n. 14004, Córdoba, Spain; CIBER Fisiopatología de la Obesidad y Nutrición (CIBEROBN), Instituto de Salud Carlos III, Madrid, Spain; Endocrinology and Nutrition Service, Hospital Virgen de la Victoria, Málaga, Spain; Department of Cell Biology, Physiology, and Immunology, IMIBIC/Reina Sofia University Hospital/University of Córdoba, Cordoba, Spain; UCD Institute of Food & Health/UCD Conway Institute, School of Public Health and Population Sciences, University College Dublin, Dublin, Ireland

**Keywords:** Metabolic syndrome, Proteomics, Inflammation, Oxidative stress, DNA damage

## Abstract

**Background:**

Metabolic syndrome is a multi-component disorder associated to a high risk of cardiovascular disease. Its etiology is the result of a complex interaction between genetic and environmental factors, including dietary habits. We aimed to identify the target proteins modulated by the long-term consumption of four diets differing in the quality and quantity of lipids in the whole proteome of peripheral blood mononuclear cells (PBMC).

**Results:**

A randomized, controlled trial conducted within the LIPGENE study assigned 24 MetS patients for 12 weeks each to 1 of 4 diets: a) high-saturated fatty acid (HSFA), b) high-monounsaturated fatty acid (HMUFA), c) low-fat, high-complex carbohydrate diets supplemented with placebo (LFHCC) and d) low-fat, high-complex carbohydrate diets supplemented with long chain (LC) n-3 polyunsaturated fatty acids (PUFA) (LFHCC n-3). We analyzed the changes induced in the proteome of both nuclear and cytoplasmic fractions of PBMC using 2-D proteomic analysis. Sixty-seven proteins were differentially expressed after the long-term consumption of the four diets. The HSFA diet induced the expression of proteins responding to oxidative stress, degradation of ubiquitinated proteins and DNA repair. However, HMUFA, LFHCC and LFHCC n-3 diets down-regulated pro-inflammatory and oxidative stress-related proteins and DNA repairing proteins.

**Conclusion:**

The long-term consumption of HSFA, compared to HMUFA, LFHCC and LFHCC n-3, seems to increase the cardiovascular disease (CVD) risk factors associated with metabolic syndrome, such as inflammation and oxidative stress, and seem lead to DNA damage as a consequence of high oxidative stress.

**Electronic supplementary material:**

The online version of this article (doi:10.1186/s12864-015-1725-8) contains supplementary material, which is available to authorized users.

## Background

Metabolic syndrome (MetS) is a multi-component disorder characterized by abdominal obesity, dyslipidaemia, hypertension and impaired insulin sensitivity, and it is associated with an increased risk of type 2 diabetes (T2DM) and cardiovascular diseases (CVD) [1, 2]. The etiology of MetS is the result of a complex interaction between genetic and environmental factors, including dietary habits, particularly the quality of dietary lipids [3].

MetS is also associated to a low-grade inflammation, which links MetS patients with atherosclerosis risk development [4]. Additionally, MetS patients have increased oxidative stress, which is directly related to the inflammatory process [5]. Reactive oxygen species (ROS) also trigger alterations in biomolecules such as lipids, proteins and DNA, which in turn, activate cellular systems to repair the proteins and DNA [6].

Diet, and particularly dietary lipids, has been shown to modulate these processes [7, 8]. In fact, monounsaturated fatty acids (MUFA)-rich diets reduce NF-κB activation compared with butter- and walnut-enriched diets or Western diets [9], and reduce the pro-inflammatory and oxidative status [10–12]. In addition, low-calorie [13] and MUFA-rich [14] diets reduce oxidative stress. Moreover, the consumption of saturated fatty acids has been shown to contribute to the development of MetS by producing pro-inflammatory cytokines such as tumor necrosis factor-α (TNF-α) and interleukin-6 via NF-kB activation [1].

Many studies use as an *in vivo* cellular model peripheral blood mononuclear cells (PBMC), a subset of white blood cells consisting of lymphocytes and monocytes/macrophages, which are easily isolated in humans from blood samples and frequently used to assess biological responses [15–17], including dietary intervention [18–20]. Most of them analyze gene expression, and while this is relevant, they have the intrinsic limitation of not focusing on the final products that perform the biological function. In fact, we have previously shown in a sub-group of the Lipgene study (which the population of this study belongs to) how an increased expression of antioxidant genes in PBMC seems to be due to the response to the postprandial oxidative stress generated mainly in adipose tissue after the consumption of an high-saturated fatty acid diet (HSFA). We have also shown how the long-term consumption of a healthy diet model with high-monounsaturated fatty acid (HMUFA) attenuates the postprandial inflammatory state and oxidative stress associated with MetS [19–21]. These gene expression changes were supported by changes in the regulatory elements at protein levels, so it may be expected that the diet would cause changes in the proteome.

A proteomic approach using two-dimensional polyacrylamide gel electrophoresis (2D-PAGE) coupled with mass spectrometry protein identification is becoming widely used as a tool for monitoring changes in protein expression which may be associated to a specific disease or therapeutic response [22], including dietary intervention [23].

We recently described the changes undergone by the proteome after the intake of four different meals in terms of lipid quality [24]. Although that study allowed us to identify fast-response proteins for dietary lipids which respond to an acute intake, it raised questions about the effect of the long-term consumption of dietary models differing in quantity and quality of lipids. Thus, in this work, we aimed to assess the changes in the proteome of MetS patients after 12 weeks’ consumption of the four dietary models, in order to identify the target proteins that are modulated in response to the quantity and quality of lipids.

## Results

### Baseline and post-intervention metabolic characteristics

No significant differences were observed in the baseline or post-intervention metabolic characteristics of the 24 subjects with MetS participating in the dietary intervention. In addition, we have not found any differences in the fasting levels of lipids and metabolic parameters after the consumption of the diets as compared with the baseline (Table 1).

**Table 1:** Baseline characteristics of 24 subjects with the MetS assigned to each diet

*Characteristics*	*HSFA*	*HMUFA*	*LFHCC*	*LFHCC n-3*	*p-value*
*Age (years)*	59.5 ± 3.9	52.5 ± 6.6	61.2 ± 5.2	54.0 ± 11.0	0.142
*BMI (kg/m* ^*2*^ *)*	36.8 ± 2.4	33.7 ± 4.3	35.2 ± 3.4	34.3 ± 3.8	0.483
*Pre-intervention*					
*Waist circumference*	118.7 ± 6.4	108.1 ± 6.4	111.7 ± 10.8	107.1 ± 7.5	0.085
*TC (mg/dl)*	193.9 ± 23.4	194.3 ± 36.5	188.0 ± 49.7	190.9 ± 34.7	0.990
*TAG total (mg/dl)*	132.0 ± 57.3	135.3 ± 54.8	128.6 ± 32.8	138.0 ± 66.9	0.992
*LDL-c (mg/dl)*	130.5 ± 24.1	141.7 ± 25.4	133.7 ± 38.9	130.3 ± 33.8	0.911
*HDL-c (mg/dl)*	44.5 ± 3.4	39.5 ± 11.4	40.4 ± 9.4	43.8 ± 8.7	0.658
*Glucose (mg/dl)*	109.4 ± 12.9	112.1 ± 14.1	102.3 ± 9.2	114.0 ± 20.0	0.536
*Insulin (mU/ml)*	18.4 ± 6.3	10.9 ± 4.5	14.5 ± 6.3	13.0 ± 5.5	0.175
*Post-intervention*					
*Waist circumference*	119.1 ± 2.9	108.5 ± 3.3	111.2 ± 4.4	108.8 ± 2.6	0.125
*TC (mg/dl)*	192.3 ± 7.9	191.3 ± 6.6	175.6 ± 15.8	172.3 ± 11.09	0.458
*TAG total (mg/dl)*	107.3 ± 27.3	136.3 ± 17.6	133.0 ± 15.8	109.5 ± 18.5	0.455
*LDL-c (mg/dl)*	132.5 ± 6.9	135.0 ± 6.7	122.6 ± 14.6	112.9 ± 10.7	0.427
*HDL-c (mg/dl)*	43.5 ± 2.1	40.1 ± 2.1	38.3.4 ± 1.8	44.6 ± 2.3	0.384
*Glucose (mg/dl)*	103.5 ± 4.8	107.1 ± 6.0	103.8 ± 10.3	107.7 ± 8.9	0.972
*Insulin (mU/ml)*	16.7 ± 2.2	15.3 ± 2.2	14.5 ± 2.9	13.5 ± 1.0	0.770

Values presented are the mean ± SEM of each diet group. One-way ANOVA statistical analysis *p-values*

### Proteomic analysis

Our proteomic 2-D PAGE analysis of the changes in the proteome of PBMC, at the level of nuclear and cytoplasmic fractions separately, after the long-term intake of the four different diets, showed an average of >250 protein spots detected in the nuclear fraction and > 350 protein spots detected in the cytoplasmic fraction.

The long-term consumption of the four diets (12-week dietary intervention) induced significant changes in the proteome of PBMC in both nuclear (Table 2; see Additional file 1: Table S1) and cytoplasmic fractions (Table 3; see Additional file 2: Table S2). In the nuclear fraction, we observed significant changes in 29 proteins. The long-term consumption of the HSFA diet induced the up-regulation of 4 proteins (CHMB1.5, ASB11, VASP, NFX1) and the down-regulation of 2 proteins (ACTB and ARPC2). The consumption of the HMUFA diet induced the up-regulation of ZSCAN29 protein and the down-regulation of 8 proteins (VPS28, TLN1, GSN, FGB, FGA, F2, POLR3E, ANX2). The consumption of LFHCC induced the up-regulation of 2 proteins, GSN and ZFP624, and the down-regulation of 8 proteins (THBS1, TPM, REV3-L, MYO1, MACF1, FGB, CAP and one hypothetical protein) while the consumption of LFHCC with n-3 fatty acids caused the up-regulation of 6 different proteins (FGG, VCL, PA2G4, MSN, PLEK, FGB) and the down-regulation of CLIC1 protein.

**Table 2:** Long-term effect of quantity and quality of dietary fat on proteome of the nuclear fraction of PBMC

***Proteomic changes Induced after 12 weeks of the intake of HSFA diet consumption***			
*Protein*	*Symbol*	*FC*	*p-Value*
*- Beta actin partial*	ACTB	0.56	0.041
*- Charged multivesicular body protein 1b*	CHMB1.5	2.64	0.038
*- Actin related protein 2/3 complex, subunit 2, 34 kDa,isoform*	ARPC2	DR	0.009
*- Ankyrin repeat and SOCS box protein 11*	ASB11	1.98	0.049
*- Vasodilator-stimulated phosphoprotein*	VASP	3.51	0.041
*- Transcriptional repressor NF-X1*	NFX1	UR	0.009
***Proteomic changes Induced after 12 weeks of the intake of HMUFA diet consumption***			
*Protein*	*Symbol*	*FC*	*p-Value*
*- Vacuolar protein sorting-associated protein 28 homolog*	VPS28	DR	0.003
*- Talin 1*	TLN1	0.50	0.036
*- Zinc finger and SCAN domain-containing protein 29*	ZSCAN29	1.83	0.031
*- Gelsolin*	GSN	0.35	0.043
*- Fibrinogen, beta chain isoform CRA e*	FGB	0.33	0.038
*- Alpha-fibrinogen precursor*	FGA	0.04	0.044
*- Chain B, Crystal Structure Of The Thrombin MutantG193p*	F2	0.53	0.015
*- DNA-directed RNA polymerase III subunit*	POLR3A	0.27	0.034
*- Annexin A2*	ANX2	0.33	0.050
***Proteomic changes Induced after 12 weeks of the intake of LFHCC diet consumption***			
*Protein*	*Symbol*	*FC*	*p-Value*
*- Thrombospondin 1 precursor*	THBS1	0.34	0.019
*- Putative tropomyosin alpha-3 chain-like protein*	TPM	0.53	0.030
*- REV3-like, catalytic subunit of DNA polymerase zeta*	REV3-L	0.21	0.001
*- Myosin-1*	MYO1	0.25	0.039
*- Microtubule-actin cross-linking factor 1*	MACF1	0.54	0.015
*- Fibrinogen beta chain*	FGB	0.40	0.045
*- Adenylate cyclase-associated protein 1 (yeast)*	CAP	DR	0.009
*- Gelsolin*	GSN	6.16	0.024
*- Hypothetical protein*		0.71	0.024
*- Zinc finger protein 624*	ZFP624	1.61	0.034
***Proteomic changes Induced after 12 weeks of the intake of LFHCC + n3 diet consumption***			
*Protein*	*Symbol*	*FC*	*p-value*
*- Fibrinogen gamma chain, isoform CRA_j*	FGG	1.88	0.048
*- Vinculin*	VCL	3.23	0.026
*- Proliferation associated 2G4 protein*	PA2G4	2.17	0.001
*- Moesin*	MSN	2.61	<0.05
*- Pleckstrin*	PLEK	1.84	0.025
*- Fibrinogen beta chain*	FGB	1.85	0.039
*- Chloride intracellular channel 1 protein*	CLIC1	DR	<0.50
*- Fibrinogen beta chain, isoform CRA_d*	FGB	4.90	<0.50

Proteins differentially expressed in the post-intervention compared to the baseline from an average of over 250 protein spots detected in the nuclear fraction. FC: Fold change. *T*-test p-value. UR, up-regulated proteins, not detected at baseline but detected at post-intervention. DR, down- regulated, proteins detected at baseline but not detected at post-intervention

**Table 3:** Long-term effect of quantity and quality of dietary fat on proteome of cytoplasmic fraction of PBMC

***Proteomic changes Induced after 12 weeks of the intake of HSFA diet consumption***			
*Protein*	*Symbol*	*FC*	*p-Value*
*- Target of Myb protein 1*	TOM1	2.06	0.027
*- DNA-directed RNA polymerase III subunit*	POLR3E	3.08	0.012
*- Chain A, X-Ray Crystal Structure Of Zinc-Bound F95mW97V CARBONIC Anhydrase (Caii) Variant*		UR	0.017
*- Coiled-coil domain-containing protein 88B. Isoform 3*	CCDC88B	DR	0.026
***Proteomic changes Induced after 12 weeks of the intake of HMUFA diet consumption***			
*Protein*	*Symbol*	*FC*	*p-Value*
*- Chloride intracellular channel 1 protein*	CLIC1	0.63	0.032
*- Protein disulfide isomerase-related protein 5*	PDIA5	0.47	0.008
*- Capping protein (actin filament) muscle Z-line, beta*	CAPZ	0.32	0.024
*- hCG1808619*		DR	0.024
*- Nuclear receptor-interacting protein 3*	NRIP3	DR	0.023
*- Zinc finger protein 791*	ZFP791	1.89	0.038
***Proteomic changes Induced after 12 weeks of the intake of LFHCC diet consumption***			
*Protein*	*Symbol*	*FC*	*p-Value*
*- hCG2041202*		0.62	0.001
*- NF-kappa-B-activating kinase*	TBK1	0.62	0.019
*- Growth factor receptor-bound protein 2 isoform 1*	GRB2	0.35	0.026
*- Sry-related HMG box gene*		0.16	0.007
*- PSMA4 protein*	PSMA4	1.81	0.071
*- Voltage-dependent anion channel 2, isoform CRA_a*	VDAC2	0.70	0.046
***Proteomic changes Induced after 12 weeks of the intake of LFHCC + n3 diet consumption***			
*Protein*	*Symbol*	*FC*	*p-Value*
*- Vacuolar protein sorting-associated protein 28 homol*	VPS28	1.85	0.040
*- BiP Protein*	BiP	1.77	0.029
*- Chloride intracellular channel 1 protein*	CLIC1	0.40	0.013
*- Beta actin*	ACTB	DR	<0.05
*- POTE-2 alpha actin*	POTE-2	0.38	0.014
*- Capping protein (actin filament) muscle Z-line, beta*	CAPZ	DR	<0.05
*- HSP70-2*	HSP70-2	0.35	0.049
*- Microtubule-actin cross-linking factor 1*	MACF1	DR	<0.05
*- S4-SRCRB*		4.05	0.009
*- Gelsolin*	GSN	1.67	0.023

Proteins differentially expressed in the post-intervention compared to the baseline from an average of over 350 protein spots detected in the cytoplasmic fraction. FC: Fold change. *T*-test p-value. UR, up-regulated proteins, not detected at baseline but detected at post-intervention. DR, down- regulated, proteins detected at baseline but not detected at post-intervention

In the cytoplasmic fraction, we observed significant changes in 25 proteins after the long-term consumption of the four diets. The long-term consumption of the HSFA diet induced the up-regulation of 3 proteins (TOM1, POLR3E and Chain A, X-Ray Crystal Structure of Zinc-Bound carbonic Anhydrase (Caii)), and the down-regulation of CCDC88B protein. The consumption of the HMUFA diet caused the up-regulation of ZFP791 protein and the down-regulation of 5 proteins (CLIC1, PDIA5, CAPZ, hCG1808619, NRIP3). The LFHCC diet induced the up-regulation of PSMA4 protein and the down-regulation of 5 proteins (hCG2041202, TBK1, GRB2, Sry-related protein, VDAC2) while the LFHCC with n-3 fatty acids caused the up-regulation of 4 proteins (VPS28, BiP, S4-SRCRB, GSN) and the down-regulation of 6 proteins (CLIC1, ACTB, POTE-2, CAPZ, HSP70-2, MACF1).

### Relationship between MetS and proteome changes

In order to evaluate the relationship between the MetS and the changes in the proteome, we performed a Pearson correlation analysis between the fold change of the MetS parameters and the fold change of the protein differentially expressed by the diets (Table 4). We observed a positive relationship in the fold change of GSN and CLIC1 levels (R = 0.871, *p* = 0.024, and R = 0.971, *p* = 0.006, respectively), and a negative relationship in the fold change of F2 and CAPZ levels (R = −0.920, *p* = 0.009, and R = −0.915, *p* = 0.030, respectively), with that of the TAG levels. In addition, we observed a positive relationship in the fold change of CAPZ and PMSA4 (R = 0.951, *p* = 0.013, and R = 0.879, *p* = 0.049, respectively), and a negative relationship in the fold change of PLEK (R = −0.820, *p* = 0.046), with that of glucose levels. We also found a positive relationship between FGA and PLEK (R = 0.894, *p* = 0.041, and R = 0.842, *p* = 0.036, respectively), and the fold change of the waist perimeter.

**Table 4:** Correlation analysis between MetS and proteome changes

*Parameter/Protein*	*FGA*	*F2*	*GSN*	*PLEK*	*CLIC1*	*CAPZ*	*PSMA4*
*Triglycerides*	−0.611	−0.920	0.871	−0.750	0.971	−0.915	−0.841
	0.197	0.009	0.024	0.086	0.006	0.030	0.075
*Glucose*	0.442	0.076	−0.624	−0.820	−0.785	0.951	0.879
	0.380	0.886	0.185	0.046	0.116	0.013	0.049
*Waist perimeter*	0.894	0.518	−0.510	0.842	−0.303	0.579	0.775
	0.041	0.371	0.301	0.036	0.697	0.421	0.124

Pearson correlation analysis between the fold change of the parameters of MetS and the fold change of the protein differentially expressed by the diets. FGA: Alpha-fibrinogen precursor, F2: Chain B, Crystal Structure Of The Thrombin MutantG193p, GSN: Gelsolin isoform c, PLEK: Pleckstrin, CLIC1: Chloride intracellular chanel 1 protein, CAPZ: Capping protein (actin filament) muscle Z-line, beta, PSMA4: PSMA4 protein

### Pathway analysis

Pathway analysis was performed by using the Ingenuity Pathway program, which allowed us to identify those metabolic pathways or possible biomechanisms that may be regulated in response to the long-term consumption of the four different diets. These biomechanisms can be grouped in 6 clusters of cellular functions: (A) *movement, proliferation and migration of cells* (ANX2, F2, FGA, GRB2, BiP, MSN, PA2G4, THBS1, TLN1, TPM1, VASP, VASP28, VCL), (B) *cellular assembly and organization* (ARPC2, F2, PLEK, TBK1, THBS1, TPM1, (C) *regulation and metabolism* (NFX1, CC88B, S100A10), (D) *chaperones and stress response* (VDAC2, HSPA1A, HSPA1B, BiP), (E) *cellular cycle* (MYO1, NFX1), (F) *DNA integrity* (REV3-L, PSMA4, POLR3E), (F) *others* (NRIP3, ZNF624, ZNF791, ZSCAN2).

**Fig. 1:**
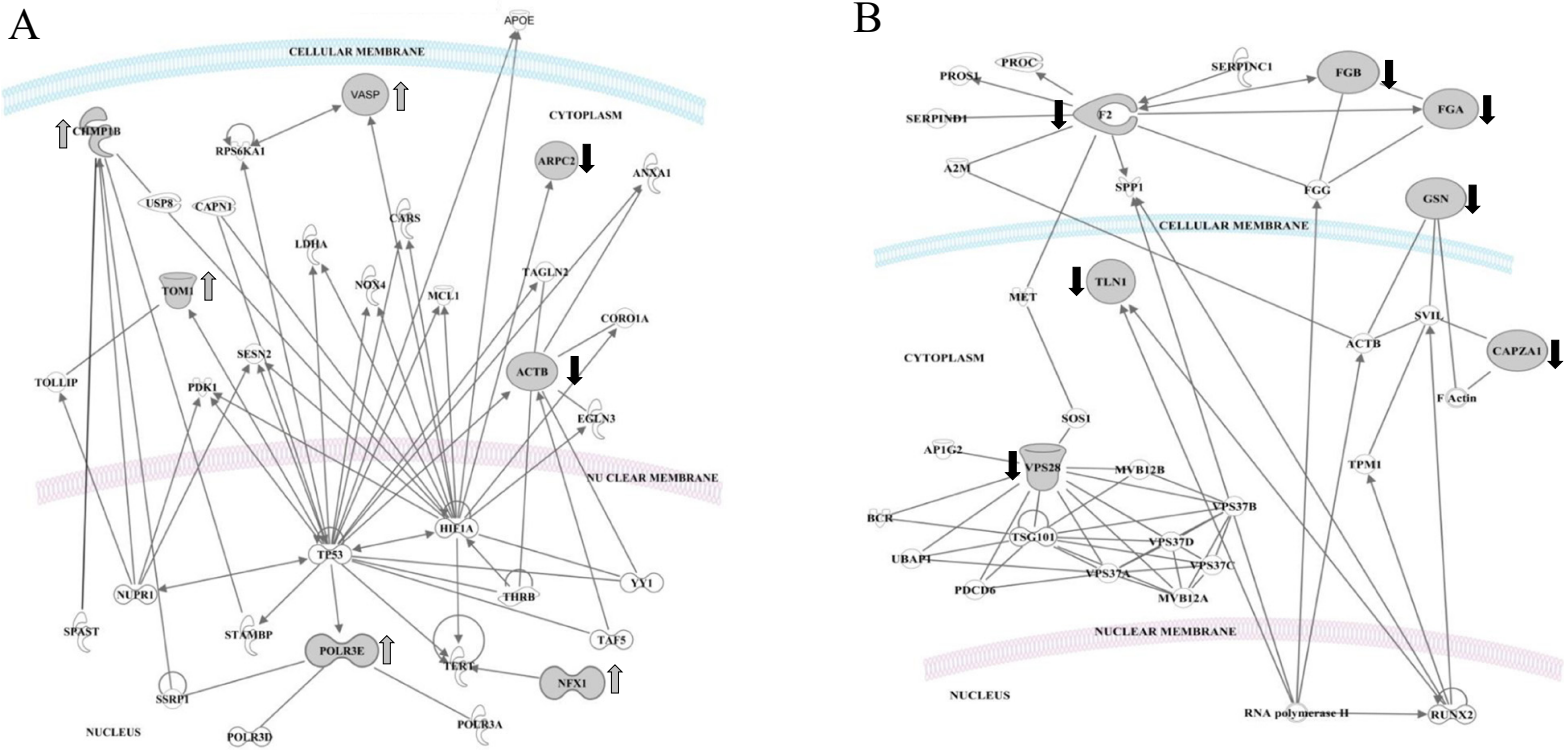
Interaction between induced proteins at the long-term consumption of (**a**) HSFA and (**b**) HMUFA diets. Networks of differentially expressed proteins in the long-term ingestion of: **a**) HSFA, Saturated fatty acid diet; **b**) HMUFA, Monounsaturated fatty acid diet

Our analysis also showed that the long-term consumption of the HSFA diet induced changes in proteins involved in five interaction networks (Fig. 1a), which are mainly related with the degradation of unfolding proteins (nuclear CHMP1.5), cell to cell signaling interaction (nuclear VASP and cytoplasmic TOM1), morphology and cardiovascular system development (nuclear ARPC2). Analysis of the biofunctions related with diseases and disorders showed that these proteins are included in neurological (*p* = 5.8E-04–1.2E-02) and infectious diseases (CHMP.5 and ACTB) (*p* = 8.5E-03 – 8.5E-03). Top canonical pathway analysis showed the interaction of three nuclear proteins ACTB, ARPC2 and VASP within the pathway Fcγ receptor-mediated phagocytosis in macrophages and monocytes [*p* = 1.55E-05; ratio 3/95 (0.032)].

The long-term consumption of the HMUFA diet induced changes in proteins involved in three different networks (Fig. 1b): developmental disorder (nuclear FGA, FGB, ACTB and GSN), DNA replication, recombination and repair (nuclear POLR3E) and inflammatory response (nuclear F2, TLN1, GSN and cytoplasmic CAPZ). The molecular and cellular functions analysis included the proteins involved in mechanisms such as cell to cell signaling (FGA, FGB, F2 and TLN1), cell function and maintenance (GSN), and cell death and survival (F2, GSN and VPS28). In terms of diseases and disorders, the biofunctions analysis showed two items at the level of hematological and immunological diseases, with five proteins inter-related (FGB, FGA, ANXA2, GSN and F2). Analysis of the canonical pathways indicates that the protein changes identified interact in pathways related with extrinsic and intrinsic prothrombin activation and the coagulation system (*p* = 8.0E-10; 9.0E-09; 2.3E-08; respectively).

**Fig. 2:**
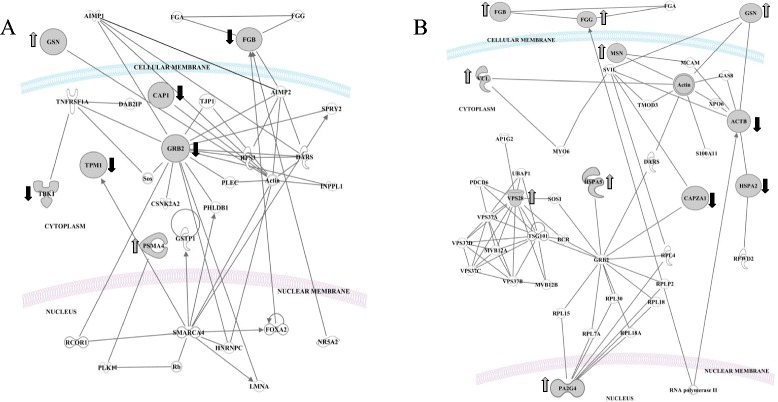
Interaction between induced proteins at the long-term consumption of (**a**) LFHCC and (**b**) LFHCC n-3 diets. Networks of differentially expressed proteins in the long-term ingestion of: **a**) LFHCC, Low fat, high-complex carbohydrate diet with placebo; **b**) LFHCC n-3, Low fat, high-complex carbohydrate diet with 1.24 g/d LC n-3 PUFA diet

Long-term consumption of the LFHCC diet showed significant changes in the proteins interlinked in one main network (Fig. 2a), that includes six molecules related with organism function (nuclear CAP, GSN, TPM, THBS1 and cytoplasmic GRB2, TBK1), tissue morphology, organism injury and abnormalities. Analysis of the molecular and cellular functions suggests that these proteins are involved in the carbohydrate metabolism, cell to cell signaling (THBS1 and GRB2) and cellular assembly and organization (nuclear GSN, CAP, THBS1, MYO1, TPM, and cytoplasmic GRB2, TBK). Our results showed that at the level of the biofunctions related with diseases and disorders, the changes induced by a chronic intake of the LFHCC diet are implicated in cardiovascular and metabolic diseases (TPM, GSN and THBS1). The canonical pathway analysis shows that the changes identified are related with actin cytoskeleton signaling (ACTB, GSN, GRB2) (*p* = 8.5E-04).

The LFHCC n-3 diet induced changes in the proteome of PBMC (Fig. 2b) related with the inflammatory response (nuclear FGB, FGG, VCL, and cytoplasmic ACTB, MACF1, CAPZA1), cellular aggregation (nuclear MSN and GSN from cytoplasmic) and cellular assembly (the cytoplasmic VPS28). Analysis of the molecular and cellular functions shows that the proteins identified after the ingestion of this diet are involved in cellular assembly and organization (the nuclear PLEK and MSN, and the cytoplasmic GSN) and cell death and survival (VPS28 and S100-A10 both in cytoplasm). Likewise, analysis of the diseases and disorders groups shows the proteins that are involved in immunological diseases and inflammatory response. Canonical pathway analysis after the long-term intake of LFHCC n-3 shows changes in the extrinsic prothrombin activation pathway associated with the proteins differentially expressed (*p* = 6.68E-05).

## Discussion

Our study showed for the first time the effect of the long term consumption of four dietary models, providing different quantity and quality of dietary lipids, on the whole proteome of peripheral blood mononuclear cells (PBMC) from patients with MetS. In this study, we analyzed separately the changes induced in the nuclear and cytoplasmic proteome after the long-term consumption of a high saturated fatty acid diet (HSFA), a high monounsaturated fatty acids-rich diet (HMUFA), a low-fat high carbohydrates diet (LFHCC) and a low-fat high carbohydrates diet supplemented with n-3 fatty acids (LFHCC n-3).

The proteins responding to the quantity and quality of dietary lipids identified in our study showed that dietary lipids regulate different biomechanisms directly involved in the etiology of MetS. This idea is supported by the relationship found between MetS parameters, mainly in changes in the TAG and glucose levels and several changes in the proteome. Long-term consumption of an HSFA diet leads to the increase of a protein involved in the degradation of ubiquitinilated proteins (nuclear CHMP1.5, also known as VSP46; and cytoplasmic TOM1), and DNA repair (POLR3E), both of which mechanisms are activated in response to the oxidative damage caused by reactive oxygen species (ROS). In contrast, HMUFA, LFHCC and LFHCC n-3 diets showed a decrease in the expression of proteins directly related with oxidative stress, inflammation, endoplasmic reticulum stress (ER) and DNA repair.

In terms of cellular function, the 56 differentially expressed proteins after the consumption of the four diets (nuclear and cytoplasmic fractions), are mainly involved in four cellular processes: (1) movement, proliferation and migration, (2) cellular assembly and organization, (3) chaperones and oxidative stress response, and (4) DNA integrity. Similarly, these cellular mechanisms are related to four biological pathways: inflammation, oxidative stress, ER stress and DNA repair.

The long term consumption of the HSFA diet up-regulated CHMP1.5 and TOM1 proteins, both involved in the protein degradation by the ubiquitination system in the ER. While CHMBP1.5 is involved in the formation of multivesicular bodies (MVB) [25], TOM1 binds ubiquitin, which suggests that these proteins may participate in the sorting of ubiquitinated proteins into MVB [26, 27]. In addition, the up-regulation after an HSFA diet of POLR3E, an oxidative stress-induced protein, is directly related with the DNA repair process after oxidative damage [28]. Moreover, a pro-inflammatory effect of HSFA consumption is suggested by the up-regulation of the vasodilatador-stimulated protein (VASP) and the down-regulation of ARPC2 and actin, both involved in the control of migration and cellular aggregation characteristic of inflammatory processes [29–31]. Additionally, the up-regulation of the inflammatory-related NFX1 by HSFA consumption reinforces the idea that oxidative stress and inflammation are processes which are related to each other [32].

In contrast, the long-term consumption of an HMUFA diet down-regulated proteins involved in oxidative stress, and prothrombotic and pro-inflammatory proteins. In fact, there was a decrease in the expression of sortin-associated protein 28 (VPS28), involved in the ubiquitination of damaged proteins by oxidative stress [33], and POLR3E, a protein involved in DNA repair after oxidative damage [34]. These results suggests that the lower DNA damage after the long-term consumption of an HMUFA diet is presumably due to lower oxidative stress after the consumption of this diet, as previously described [20, 35]. In keeping with this, a previous study by our group demonstrated in the same population as this study an imbalance in the cellular redox state or oxidative stress after an HSFA diet, with a reduction in oxidative stress after HMUFA diet consumption [20].

In addition, the consumption of the HMUFA diet also down-regulated seven proteins involved in thrombus formation: prothrombin or coagulation factor II (F2), fibrinogen (FGB), alpha-fibrinogen precursor (FGA), Talin1 (TLN1) Gelsolin (GSN), Annexin2 (ANX2) and Capping protein (actin filament) muscle Z-line, beta (CAPZA1), which is especially important in MetS patients, as they are characterized by a pro-thrombotic state [36]. In fact, high FGB levels are associated with an increased risk of coronary artery disease and the pathogenesis of myocardial infarction [37], and TLN1 participates in the establishment of focal cell to cell adhesions, which are characteristic in the pro-thrombotic and inflammatory process [38]. On the other hand, ANX2 and the capping protein actin (CAP) are involved in the activation of macrophages [39–41], and gelsolin (GSN) in phagocytosis [42].

Both of the low-fat diets were characterized by a lower activation of the inflammatory pathway. The changes in the proteome observed after an HSFA diet support the idea of an increased production of reactive oxygen species, whereas changes in the proteome induced by HMUFA reduced the risk of plaque formation of atheroma and reduced oxidative stress.

This is based on the down-regulation of TBK1, THBS-1, FGB, TPM1, ACTB, and MYO1 proteins after LFHCC consumption. TBK1 is a member of the IκB kinase (IKK) family and plays an important role in the regulation of the immune response [43, 44], THBS-1 has been shown to promote platelet aggregation [45], and fibrinogen beta chain (FGB) protein, the substrate of the limiting step in the production of mature fibrinogen which is cleaved by thrombin to form insoluble fibrin, the most abundant component of blood clots [46]. Proteins such as actin (ACTB), myosin (MYO) and tropomyosin (TPM) interact to form the extracellular matrix and provide stability to the atherosclerotic plaque [47].

Moreover, the long-term consumption of both an LFHCC diet and the HMUFA diet down-regulated the REV3L protein, which participates actively in the repair of DNA damage and is induced by a variety of stimuli, including oxidative stress [48]. These results support the idea that the long-term consumption of an LFHCC diet reduces oxidative stress [20], which may prevent DNA damage by ROS, as previously reported [35, 49]. Thus, the long-term consumption of LFHCC n-3 diet down-regulated proteins involved in cellular mechanisms such as oxidative stress, inflammation and endoplasmic reticulum function. Recent studies have demonstrated that chloride intracellular channel 1 (CLIC1) has a binding site for glutathione (GSH) in its N-terminal domain and plays an important role in the generation of ROS via the NADPH-oxidase system, participating in the channel ion exchange required for the synthesis of ROS [50]. Moreover, the long-term consumption of an LFHCC n-3 diet also down-regulated S100-A10 protein, which forms a complex with annexin2, a mediator of macrophage activation in the inflammatory response [51]. We also observed a down-regulation in MACF1 protein after LFHCC n-3 diet consumption. This protein belongs to the family of plakins and plays a role in the cytoskeletal dynamic that is necessary for cell migration and chemotaxis, which are typical in mechanisms such as inflammation [52, 53]. Additionally, at ER level, the up-regulation of BiP, an ER stress sensor protein [54], and the down-regulation of HSP70, a protein involved in the transport and folding of proteins in response to oxidative damage, suggest a modulation of ER stress following the consumption of LFHCC n-3.

One limitation of this study is that, although we are able to identify protein profile changes indicative of the modulations of the CVD risk factors associated with MetS, further studies will be needed in order to work out how to categorize diets according to their efficiency as anti-inflammatory or antioxidant treatments, based on the changes induced on the whole proteome.

## Conclusion

The long term consumption of HSFA, compared to HMUFA, LFHCC and LFHCC n-3, may increase the CVD risk factors associated with metabolic syndrome, such as inflammation and oxidative stress, in addition to the DNA damage which is a consequence of high oxidative stress. The implications of this study for the general nutrition of the population, and specifically for MetS patients, lie in the adaptive effect observed after the long-term consumption of four dietary models differing in the quantity and quality of dietary lipids.

## Methods

### Participants and recruitment

We performed the current study within the framework of the LIPGENE study (Diet, genomics and metabolic syndrome: an integrated nutrition, agro-food, social and economic analysis; Clinical Trial Registration Number: NCT00429195 (Study identifier at ClinicalTrials.gov)), a Framework 6 Integrated Projected funded by the European Union. All participants gave their written informed consent and underwent a comprehensive medical history, physical examination and clinical chemical analysis before enrolment. This study was carried out in the Lipid and Atherosclerosis Unit of the Reina Sofia University Hospital, from February 2005 to April 2006. The experimental protocol was approved by the Reina Sofia University Hospital Ethics Committee, according to the Helsinki Declaration.

We analyzed the PBMC of a subgroup (24 MetS patients, 6 patients per diet, 3 women and 3 men) from the LIPGENE Córdoba cohort, using 2-D proteomic analysis. Sample size calculation was based on previous studies in nutrition that examined the effects on the whole proteome [23, 24].

### Design, Randomization and intervention

The MetS patients were randomly stratified into 1 of 4 dietary models. MetS was defined by published criteria [55], which conformed to the LIPGENE inclusion and exclusion criteria [56]. Randomization was completed centrally according to age, gender and fasting plasma glucose concentration, using the Minimization Program for Allocating Patients to Clinical Trials randomization program (Department of Clinical Epidemiology, London Hospital Medical College, UK).

The intervention study design and intervention protocol, which also provides information about pre-, mid-, and post-intervention food consumption and dietary compliance, have been described in detail by Shaw et al. [56]. The dietary targets of the four isoenergetic diets was as follows: the high saturated fatty acid (HSFA) diet provided 38 % of total energy (E): 16 % SFA, 12 % MUFA and 6 % PUFA; the high monounsaturated fatty acid (HMUFA) diet provided 38 % of total E: 8 % SFA, 20 % MUFA and 6 % PUFA; the low-fat high-complex carbohydrate (LFHCC) diet provided 28 % of total E: 8 % SFA 11 % MUFA and 6 % PUFA with 1 g/d high oleic sunflower oil (placebo); and the low-fat, high-complex carbohydrate diet (LFHCC n-3) provided 28 % of total E: 8 % SFA, 11 % MUFA, 6 % PUFA with 1.24 g/day long-chain n-3 polyunsaturated fatty acids [19]. Participants ingested the diet for which they were randomized over a period of 12 weeks (Fig. 1), and the food intake and health status of each participant were controlled by the medical team. The dietary intake and compliance were assessed by 3-d (2 weekdays and 1 weekend day) weighed food intake assessments at baseline, wk 6, and wk 12. The composition of the diet at the end of intervention period, as well as the targets, have been previously described [20]. Blood samples were taken on day 0 and after 12 weeks (day 84). The patients arrived at the clinical center at 08:00 h, following a 12-h fast in which they refrained from smoking; they had also abstained from alcohol intake during the preceding 7 d.

### Biochemical determinations of metabolic parameters

Lipid parameters were assessed with the modular autoanalyzer DDPPII Hitachi (Roche, Basel, Switzerland), using specific reagents (Boehringer-Mannheim, Mannheim, Germany). Determinations of total cholesterol (TC) and triglycerides (TAG) levels were made by colorimetric enzymatic methods [57, 58]; of high density lipoprotein-cholesterol (HDL-c) by colorimetric assay [59]; of low density lipoprotein-cholesterol (LDL-c) by the Friedewald formula based on CT, TAG, and HDL-c values [60]. Plasma glucose concentrations were measured with a Hitachi 917 analyzer (Boehringer Mannheim, Mannheim, Germany) by the glucose oxidase method (GOD-PAP). Plasma insulin concentrations were measured by microparticle enzyme immunoassay (Abbott Diagnostics, Matsudo-shi, Japan). Non-esterified fatty acid concentrations were measured by enzymatic colorimetric assay (Roche Diagnostics, Penzberg, Germany).

### Isolation of peripheral blood mononuclear cells

Blood was collected in tubes containing 1 g of ethylene diamine tetraacetic acid (EDTA)/L. The blood samples were diluted 1:1 in phosphate saline buffer (PBS), and the cells were isolated using Ficoll gradient by centrifugation with Lymphoprep (Axis-Shield, Dundee, Scotland) at 800 x *g* for 20 min at 20 °C. The peripheral blood mononuclear cells were collected, washed with PBS and resuspended in buffer A [mmol/L: 10 N-2 hydroxyethyl piperazine-N´-2-ethanesulfonic acid (HEPES) pH 7.5, 10 KCl, 0.1 ethylene glycol tetraacetic acid (EGTA), 0.1 EDTA, 1 dithiothreitol (DTT), 0.5 phenylmethylsulfonyl fluoride (PMSF); and cOmplete protease inhibitor cocktail Cat. No. 11697498001 (Roche Applied Science, Germany)] at −80 °C prior to protein extraction.

### Protein isolation from peripheral blood mononuclear cells

Cytoplasmic and nuclear fractions of protein from PBMC were isolated following the protocol described by Hernandez-Presa et al. [61]. The samples were thawed in ice and mixed vigorously in a vortex and were then centrifuged at 15.000 x *g*, 5 min, at 4 °C. After centrifugation, the supernatant was collected (protein cytoplasmic fraction). The pellet was resuspended in 100 μL of lysis buffer C (mmol/L: 20 HEPES pH 7.5, 400 NaCl, 1 EDTA, 1 EGTA, 1 DTT, 1 PMSF; and cOmplete protease inhibitor cocktail) and incubated for 20 min in ice, during which period the samples were mixed vigorously in a vortex every 5 min for 30 s. The protein nuclear fraction was recovered from the supernatant after centrifugation at 10.000 x *g*, 5 min, at 4 °C. For protein quantification, we used the Bradford method using Dye Reagent Protein (Bio-Rad Laboratories, Inc., Hercules, CA, USA), following the manufacturer’s instructions.

### Gel electrophoresis 2-D, imaging acquisition and spot detection

Two-dimensional polyacrylamide gel electrophoresis was performed as described by Görg et al. [62]. Briefly, 200 μg of protein were diluted in 200 μl of Rehydratation buffer (8 M urea, 2 % CHAPS, 50 mM DTT, 0.2 % Bio-Lyte 3/10 ampholyte and 0.002 % bromophenol blue). Conditions of IEF (isoelectric focussing, first dimension) and electrophoresis SDS-PAGE (second dimension) were performed similarly, as previously described [24]. Gel staining was performed overnight in darkness with SYPRO Ruby (Bio-Rad Laboratories, Inc., Hercules, CA, USA) after protein fixing using 40 % methanol and 10 % acetic acid for 2 h. The gels were washed in 40 % methanol and 10 % acetic acid twice for 1 h and once in distilled water for 30 min. The gels were visualized with UV light using ChemiDoc XRS System (Bio-Rad Laboratories, Inc., Hercules, CA, USA). Images were acquired using Quantity One 16.0 software (Bio-Rad Laboratories, Inc., Hercules, CA, USA). The spots were detected using PDQuest 8.0.1 software (Bio-Rad Laboratories, Inc., Hercules, CA, USA). The normalization method chosen was total quantity in valid spots, and we used the detection parameters previously described [24].

### MALDI-TOF MS analysis

The spots were excised automatically in a ProPic station (Genomic Solutions, Huntingdon, UK) and subjected to MS analysis. For MALDI-TOF-MS analysis, gel specimens were unstained twice (30 min, 37 °C) with 200 mM ammonium bicarbonate/40 % acetonitrile. Gel pieces, dehydrated for 5 min with pure acetonitrile and dried out for 4 h, were automatically digested with trypsin according to the standard protocols in a ProGest station (Genomic Solutions). MS and MS/MS peptide analyses of each sample were taken in a 4700 Proteomics Station (Applied Biosystems, CA, USA). Mass spectrometry was performed at the Proteomics Facility (SCAI) of the University of Cordoba, which is Node 6 of the ProteoRed Consortium financed by Genoma España and belongs to the Andalusian Platform for Genomics, Proteomics and Bioinformatics.

### Pathway analysis

In order to investigate functional relationships in the set of differentially expressed proteins, we used the Ingenuity Pathway Analysis Software version 12.0 (Ingenuity Systems, Redwood City, CA USA) [63], containing a predefined data base which curates information on over 10,000 human genes.

We performed analyses on three groups of proteins: 1) all proteins included in the first list of proteins that change significantly, regardless of the diet and the fraction in which they were identified; 2) four lists of proteins were created, one for each diet, independent of the fraction in which these proteins were identified; 3) all proteins identified in the nuclear fraction and other proteins identified in the cytoplasm fraction, both independent of the diet. Core analysis was performed using the Ingenuity Knowledge Base as a reference set of the gene population, to consider to p-value calculations. We analyzed direct and indirect interactions using all the data sources of the ingenuity pathway and only for humans.

### Statistical analysis

2-D gel analysis was performed using PDQuest software (Bio-Rad Laboratories), version 8.0. Manual corrections were also performed to validate the matches automatically generated by the software. Spot volume values were normalized in each gel by dividing the raw quantity of each spot by the total volume of all valid spots included in the same gel. Other normalizations provided by the PDQuest software were also performed with similar results. Statistical analysis was performed using PASW Statistics, Version 18 (Chicago, IL, USA). The normal distribution of variables to characterize differences in the expression of proteins under study was assessed using the Kolmogorov-Smirnov test followed by a Student’s *t*-test for independent samples. Differences were considered significant at p < 0.05. All data are expressed as mean ± S.E.M.

## Additional files

Additional file 1: Table S1. Quantity and quality dietary fat long-term effect on the proteome of the nuclear fraction of PBMC. Proteins differentially expressed in the post-intervention compared to the baseline from an average of over 250 protein spots detected in the nuclear fraction. FC: Fold change. *T*-test p-value. UR, up-regulated proteins, not detected at baseline but detected at post-intervention. DR, down- regulated, proteins detected at baseline but not detected at post-intervention. MW: Molecular weight. pI: Isoelectric point. SCI: Score C.I %. TI C.I: Total Ion C.I. %. Pep. Count: peptide count. C.I.: Confidence index. Additional file 2: Table S2. Quantity and quality dietary fat long-term effect on the proteome of cytoplasmic fraction of PBMC. Proteins differentially expressed in the post-intervention compared to the baseline from an average of over 350 protein spots detected in the cytoplasmic fraction. FC: Fold change. *T*-test p-value. UR, up-regulated proteins, not detected at baseline but detected at post-intervention. DR, down- regulated, proteins detected at baseline but not detected at post-intervention. MW: Molecular weight. pI: Isoelectric point. SCI: Score C.I %. TI C.I: Total Ion C.I. %. Pep. Count: peptide count. C.I.: Confidence index.

## Abbreviations

HSFA: High saturated fatty acid diet
HMUFA: High monounsaturated fatty acid diet
PUFA: Polyunsaturated fatty acids
LFHCC: Low fat, high-complex carbohydrate diet with placebo
LC: Long chain
LFHCC n-3: Low fat, high-complex carbohydrate diet with 1.24 g/d LC n-3 PUFA diet
PBMC: Peripheral blood mononuclear cells
MetS: Metabolic syndrome
T2DM: Type 2 diabetes
CVD: Cardiovascular diseases
ROS: Reactive oxygen species
TNF-a: Tumor necrosis factor-a
2D-PAGE: Two-dimensional polyacrylamide gel electrophoresis
ER: Endoplasmic reticulum stress
BMI: Body mass index
TC: Total cholesterol
TAG: Triglycerides
HDL-c: High density lipoprotein-cholesterol
LDL-c: Low density lipoprotein-cholesterol; energy (E)
IEF: Isoelectric focusing
EDTA: Ethylene diamine tetraacetic acid
HEPES: N-2 hydroxyethyl piperazine-N´-2-ethanesulfonic acid
EGTA: Ethylene glycol tetraacetic acid
DTT: Dithiothreitol
PMSF: Phenylmethylsulfonyl fluoride
