# Supplementary material for: Proteome from patients with metabolic syndrome is regulated by quantity and quality of dietary lipids
Source: BMC Genomics. 2015 Jul 8;16(1):509. doi: 10.1186/s12864-015-1725-8 (PMC4493955; doi:10.1186/s12864-015-1725-8)
Supplement: Additional file 1: Table S1. — Quantity and quality dietary fat long-term effect on the proteome of the nuclear fraction of PBMC. Proteins differentially expressed in the post-intervention compared to the baseline from an average of over 250 protein spots detected in the nuclear fraction. FC: Fold change. T-test p-value. UR, up-regulated proteins, not detected at baseline but detected at post-intervention. DR, down- regulated, proteins detected at baseline but not detected at post-intervention. MW: Molecular weight. pI: Isoelectric point. SCI: Score C.I %. TI C.I: Total Ion C.I. %. Pep. Count: peptide count. C.I.: Confidence index. [file 12864_2015_1725_MOESM1_ESM.docx]

**Supplemental table 1. Quantity and quality dietary fat long-term effect on the proteome of the nuclear fraction of PBMC.**

***Proteomic changes Induced after 12 weeks of the intake of HSFA diet consumption***

| ***Protein*** | ***Symbol*** | ***MW*** | ***pI*** | ***Accession No.*** | ***SCI %*** | ***Pep.***  ***Count*** | ***FC*** | ***p-Value*** |
| --- | --- | --- | --- | --- | --- | --- | --- | --- |
|  |  |  |  |  |  |  |  |  |
| *- Beta actin partial* | **ACTB** | 32.3 | 5.20 | GB:ADX77906.1 | 100 | 4 | 0.56 | 0.041 |
| *- Charged multivesicular body*  *protein 1b* | **CHMB1.5** | 21.8 | 7.80 | GB:AAG01449.1 | 100 | 5 | 2.64 | 0.038 |
| *- Actin related protein 2/3 complex, subunit 2, 34kDa,isoform b* | **ARPC2** | 21.9 | 9.10 | GB:EAW70592.1 | 100 | 5 | DR | 0.009 |
| *- Ankyrin repeat and SOCS box*  *protein 11* | **ASB11** | 34.1 | 8.40 | SWP:Q8WXH4.1 | 0 | 7 | 1.98 | 0.049 |
| *- Vasodilator-stimulated phosphoprotein* | **VASP** | 40.6 | 8.80 | SWP:P50552.3 |  | 8 | 3.51 | 0.041 |
| *- Transcriptional repressor NF-X1* | **NFX1** | 118.5 | 8.70 | SWP: Q12986.2 | 0 | 12 | UR | 0.009 |
|  |  |  |  |  |  |  |  |  |

***Proteomic changes Induced after 12 weeks of the intake of HMUFA diet consumption***

| ***Protein*** | ***Symbol*** | ***MW*** | ***pI*** | ***Accession No.*** | ***SCI %*** | ***Pep.***  ***Count*** | ***FC*** | ***p-Value*** |
| --- | --- | --- | --- | --- | --- | --- | --- | --- |
|  |  |  |  |  |  |  |  |  |
| *- Vacuolar protein sorting-associated protein 28 homolog* | **VPS28** | 24.7 | 5.30 | SWP: [Q9UK41](http://www.uniprot.org/uniprot/Q9UK41) | 90.4 | 7 | DR | 0.003 |
| *- Talina 1* | **TLN1** | 41.7 | 6.00 | GB: AAH42923.1 | 100 | 10 | 0.50 | 0.036 |
| *- Zinc finger and SCAN domain-containing protein 29* | **ZSCAN29** | 83.9 | 6.80 | SWP: Q8IWY8.2 | 80.1 | 12 | 1.83 | 0.031 |
| *- Gelsolin* | **GSN** | 72.5 | 8.00 | SWP: [P06396](http://www.uniprot.org/uniprot/P06396) | 100 | 9 | 0.35 | 0.043 |
| *- Fibrinogen, beta chain isoform CRA e* | **FGB** | 56.6 | 8.50 | SWP: [D3DP13](http://www.uniprot.org/uniprot/D3DP13) | 100 | 13 | 0.33 | 0.038 |
| *- Alpha-fibrinogen precursor* | **FGA** | 70.2 | 8.30 | GB: AAA52426.1 | 100 | 13 | 0.04 | 0.044 |
| *- Chain B, Crystal Structure Of The Thrombin MutantG193p* | **F2** | 30.2 | 8.90 | PDB: 1Z8J_B | 90.2 | 8 | 0.53 | 0.015 |
| *- DNA-directed RNA polymerase III subunit* | **POLR3A** | 99.1 | 9.50 | SWP: [O14802](http://www.uniprot.org/uniprot/O14802) | 96.2 | 10 | 0.27 | 0.034 |
| *- Annexin A2* | **ANX2** | 40.7 | 8.40 | GB: AAH66955.2 | 100 | 25 | 0.33 | 0.050 |

***Proteomic changes Induced after 12 weeks of the intake of LFHCC diet consumption***

| ***Protein*** | ***Symbol*** | ***MW*** | ***pI*** | ***Accession No.*** | ***SCI %*** | ***Pep.***  ***Count*** | ***FC*** | ***p-Value*** |
| --- | --- | --- | --- | --- | --- | --- | --- | --- |
|  |  |  |  |  |  |  |  |  |
| *- Thrombospondin 1 precursor* | **THBS1** | 133.3 | 4.70 | SWP:[Q9NS62](http://www.uniprot.org/uniprot/Q9NS62) | 100 | 6 | 0.34 | 0.019 |
| *- Putative tropomyosin alpha-3 chain-like protein* | **TPM** | 27.4 | 4.70 | SWP:A6NL28.2 | 99.2 | 5 | 0.53 | 0.030 |
| *- REV3-like, catalytic subunit of DNA polymerase zeta* | **REV3-L** | 37.4 | 9.58 | GB: EAW48292.1 | 99.0 | 10 | 0.21 | 0.001 |
| *- Myosin-1* | **MYO1** | 22.4 | 5.60 | SWP: P12882.3 | 99.9 | 24 | 0.25 | 0.039 |
| *Microtubule-actin cross-linking factor 1* | **MACF1** | 180.3 | 5.86 | SWP: Q6IPG6 | 99.7 | 40 | 0.54 | 0.015 |
| *- Fibrinogen beta chain* | **FGB** | 51.4 | 8.00 | GB: AAI07767.1 | 100 | 12 | 0.40 | 0.045 |
| *- Adenylate cyclase-associated protein 1 (yeast)* | **CAP** | 20.0 | 7.00 | SWP:[Q01518](http://www.uniprot.org/uniprot/Q01518) | 99.9 | 3 | DR | 0.009 |
| *- Gelsolin* | **GSN** | 72.5 | 8.00 | SWP: [P06396](http://www.uniprot.org/uniprot/P06396) | 100 | 9 | 6.16 | 0.024 |
| *- Hypothetical protein* |  | 98.2 | 8.90 | GB: CAE45960.2 | 98.7 | 15 | 0.71 | 0.024 |
| *- Zinc finger protein 624* | **ZFP624** | 102.5 | 9.10 | SWP:Q9P2J8.3 | 85.1 | 12 | 1.61 | 0.034 |
|  |  |  |  |  |  |  |  |  |

***Proteomic changes Induced after 12 weeks of the intake of LFHCC+n3 diet consumption***

| ***Protein*** | ***Symbol*** | ***MW*** | ***pI*** | ***Accession No.*** | ***SCI %*** | ***Pep.***  ***Count*** | ***FC*** | ***p-Value*** |
| --- | --- | --- | --- | --- | --- | --- | --- | --- |
|  |  |  |  |  |  |  |  |  |
| *- Fibrinogen gamma chain, isoform CRA_j* | **FGG** | 48.3 | 6.02 | GB: EAX04915.1 | 100 | 18 | 1.88 | 0.048 |
| *- Vinculin* | **VCL** | 117.2 | 5.80 | GB: AAH39174.1 | 100 | 19 | 3.23 | 0.026 |
| *- Proliferation associated 2G4 protein* | **PA2G4** | 42.0 | 7.10 | GB: AAH32111.1 | 100 | 9 | 2.17 | 0.001 |
| *- Moesin* | **MSN** | 67.9 | 6.10 | SWP:[P26038](http://www.uniprot.org/uniprot/P26038) | 100 | 30 | 2.61 | < 0.05 |
| *- Pleckstrin* | **PLEK** | 40.4 | 8.70 | GB:CAG46876.1 | 99.9 | 2 | 1.84 | 0.025 |
| *- Fibrinogen beta chain* | **FGB** | 51.4 | 8.00 | GB: AAI07767.1 | 100 | 17 | 1.85 | 0.039 |
| *- Chloride intracellular channel 1 protein* | **CLIC1** | 27.3 | 5.10 | SWP:[O00299](http://www.uniprot.org/uniprot/O00299) | 100 | 11 | DR | < 0.50 |
| *- Fibrinogen beta chain, isoform CRA_d* | **FGB** | 52.8 | 8.30 | GB: EAX04933.1 | 100 | 10 | 4.90 | < 0.50 |
|  |  |  |  |  |  |  |  |  |

***MW:*** *Molecular weight*

***pI:*** *Isoelectric point*

***SCI %:*** *Ion score values correspond to MASCOT scores*

***FC:*** *Fold change indicates the average volume ratio ( post- versus pre-intervention) of six independent subjects.*

***p-value:*** *p-values of repeated measures ANOVA; p<0.05.*

***DR:*** *Proteins down-regulated. Proteins that diminish their concentration to undetectable levels.*

***UR:*** *Proteins up-regulated. Proteins that increase their concentration from undetectable levels.*
