# Supplementary material for: Proteome from patients with metabolic syndrome is regulated by quantity and quality of dietary lipids
Source: BMC Genomics. 2015 Jul 8;16(1):509. doi: 10.1186/s12864-015-1725-8 (PMC4493955; doi:10.1186/s12864-015-1725-8)
Supplement: Additional file 2: Table S2. — Quantity and quality dietary fat long-term effect on the proteome of cytoplasmic fraction of PBMC. Proteins differentially expressed in the post-intervention compared to the baseline from an average of over 350 protein spots detected in the cytoplasmic fraction. FC: Fold change. T-test p-value. UR, up-regulated proteins, not detected at baseline but detected at post-intervention. DR, down- regulated, proteins detected at baseline but not detected at post-intervention. MW: Molecular weight. pI: Isoelectric point. SCI: Score C.I %. TI C.I: Total Ion C.I. %. Pep. Count: peptide count. C.I.: Confidence index. [file 12864_2015_1725_MOESM2_ESM.docx]

**Supplemental Table 2. Quantity and quality dietary fat long-term effect on the proteome of cytoplasmic fraction of PBMC.**

***Proteomic changes Induced after 12 weeks of the intake of HSFA diet consumption***

| ***Protein*** | ***Symbol*** | ***MW*** | ***pI*** | ***Accession No.*** | ***SCI %*** | ***Pep.***  ***Count*** | ***FC*** | ***p-Value*** |
| --- | --- | --- | --- | --- | --- | --- | --- | --- |
|  |  |  |  |  |  |  |  |  |
| ***-*** *Target of Myb protein 1* | **TOM1** | 38.5 | 5.68 | SWP:O60784.2 | 0 | 5 | 2.06 | 0.027 |
| ***-*** *DNA-directed RNA polymerase III subunit* | **POLR3E** | 89.1 | 7.90 | SWP: Q9NVU0.1 | 86.1 | 11 | 3.08 | 0.012 |
| ***-*** *Chain A, X-Ray Crystal Structure Of Zinc-Bound F95mW97V CARBONIC Anhydrase (Caii) Variant* |  | 29.1 | 6.87 | PDB: 1FQL_A | 99.9 | 8 | UR | 0.017 |
| ***-*** *Coiled-coil domain-containing protein 88B. Isoform 3* | **CCDC88B** | 46.5 | 6.65 | SWP: A6NC98.1 | 98.7 | 11 | DR | 0.026 |
|  |  |  |  |  |  |  |  |  |

***Proteomic changes Induced after 12 weeks of the intake of HMUFA diet consumption***

| ***Protein*** | ***Symbol*** | ***MW*** | ***pI*** | ***Accession No.*** | ***SCI %*** | ***Pep.***  ***Count*** | ***FC*** | ***p-Value*** |
| --- | --- | --- | --- | --- | --- | --- | --- | --- |
|  |  |  |  |  |  |  |  |  |
| ***-*** *Chloride intracellular channel 1 protein* | **CLIC1** | 27.3 | 5.10 | SWP:[O00299](http://www.uniprot.org/uniprot/O00299) | 100 | 7 | 0.63 | 0.032 |
| ***-*** *Protein disulfide isomerase-related protein 5* | **PDIA5** | 46.5 | 5.00 | GB: AAB50217.1 | 100 | 9 | 0.47 | 0.008 |
| ***-*** *Capping protein (actin filament) muscle Z-line, beta* | **CAPZ** | 34.2 | 6.00 | SWP:[B1AK87](http://www.uniprot.org/uniprot/B1AK87) | 100 | 9 | 0.32 | 0.024 |
| ***-*** *hCG1808619* |  | 25.7 | 4.50 | GB: EAW61140.1 | 14.6 | 5 | DR | 0.024 |
| ***-*** *Nuclear receptor-interacting protein 3* | **NRIP3** | 27.7 | 8.80 | SWP: Q9NQ35.1 | 87.3 | 8 | DR | 0.023 |
| ***-*** *Zinc finger protein 791* | **ZFP791** | 69.1 | 9.40 | SWP:[Q5REI6](http://www.uniprot.org/uniprot/Q5REI6) | 99.9 | 15 | 1.89 | 0.038 |
|  |  |  |  |  |  |  |  |  |

***Proteomic changes Induced after 12 weeks of the intake of LFHCC diet consumption***

| ***Protein*** | ***Symbol*** | ***MW*** | ***pI*** | ***Accession No.*** | ***SCI %*** | ***Pep.***  ***Count*** | ***FC*** | ***p-Value*** |
| --- | --- | --- | --- | --- | --- | --- | --- | --- |
|  |  |  |  |  |  |  |  |  |
| ***-*** *hCG2041202* |  | 26.9 | 5.90 | GB: EAX06892.1 | 50.8 | 7 | 0.62 | 0.001 |
| ***-*** *NF-kappa-B-activating kinase* | **TBK1** | 84.2 | 6.30 | SWP:[Q9UHD2](http://www.uniprot.org/uniprot/Q9UHD2) | 66.2 | 11 | 0.62 | 0.019 |
| ***-*** *Growth factor receptor-bound protein 2 isoform 1* | **GRB2** | 25.3 | 5.90 | SWP:[P62993](http://www.uniprot.org/uniprot/P62993) | 100 | 8 | 0.35 | 0.026 |
| ***-*** *Sry-related HMG box gene* |  | 38.9 | 6.50 | GB: AAB49537.1 | 93.2 | 9 | 0.16 | 0.007 |
| ***-*** *PSMA4 protein* | **PMSA4** | 29.6 | 7.60 | GB: AAH22817.2 | 54.5 | 8 | 1.81 | 0.071 |
| ***-*** *Voltage-dependent anion channel 2, isoform CRA_a* | **VDAC2** | 35.0 | 8.20 | GB: EAW54569.1 | 100 | 7 | 0.70 | 0.046 |

***Proteomic changes Induced after 12 weeks of the intake of LFHCC-n3 diet consumption***

| ***Protein*** | ***Symbol*** | ***MW*** | ***pI*** | ***Accession No.*** | ***SCI %*** | ***Pep.***  ***Count*** | ***FC*** | ***p-Value*** |
| --- | --- | --- | --- | --- | --- | --- | --- | --- |
|  |  |  |  |  |  |  |  |  |
| *- Vacuolar protein sorting-associated protein 28 homolog* | **VPS28** | 24.7 | 5.30 | SWP:Q9UK41 | 0 | 0 | 1.85 | 0.040 |
| *- BiP Protein* | **BiP** | 71.0 | 5.23 | GB: AAF13605.1 | 100 | 20 | 1.77 | 0.029 |
| *- Chloride intracellular channel 1 protein* | **CLIC1** | 27.3 | 5.10 | SWP:[O00299](http://www.uniprot.org/uniprot/O00299) | 100 | 11 | 0.40 | 0.013 |
| *- Beta actin* | **ACTB** | 41.3 | 5.56 | GB: AAH08633.1 | 100 | 6 | DR | < 0.05 |
| *- POTE-2 alpha actin* | **POTE-2** | 122.3 | 5.86 | GB: ABP57734.1 | 99.9 | 10 | 0.38 | 0.014 |
| *- Capping protein (actin filament) muscle Z-line, beta* | **CAPZ** | 34.2 | 6.00 | SWP:[B1AK87](http://www.uniprot.org/uniprot/B1AK87) | 100 | 7 | DR | < 0.05 |
| *- HSP70-2* | **HSP70-2** | 70.2 | 5.40 | GB:AAD21815.1 | 99.9 | 6 | 0.35 | 0.049 |
| *- Microtubule-actin cross-linking factor 1* | **MACF1** | 180.3 | 5.86 | SWP: Q6IPG6 | 100 | 42 | DR | < 0.05 |
| *- S4-SRCRB* |  | 15.1 | 6.04 | SWP:Q8WTU2.1 | 80.9 | 6 | 4.05 | 0.009 |
| *- Gelsolin* | **GSN** | 72.5 | 8.00 | SWP: P06396 | 100 | 6 | 1.67 | 0.023 |
|  |  |  |  |  |  |  |  |  |

***MW:*** *Molecular weight*

***pI:*** *Isoelectric point*

***SCI %:*** *Ion score values correspond to MASCOT scores*

***FC:*** *Fold change indicates the average volume ratio ( post- versus pre-intervention) of six independent subjects.*

***p-value:*** *p-values of repeated measures ANOVA; p<0.05.*

***DR:*** *Proteins down-regulated. Proteins that diminish their concentration to undetectable levels.*

***UR:*** *Proteins up-regulated. Proteins that increase their concentration from undetectable levels.*
